# Identification and clinical validation of endoplasmic reticulum genes related to pulmonary tuberculosis

**DOI:** 10.1038/s41598-025-29599-7

**Published:** 2025-12-07

**Authors:** Min Li, Yuxiu Wang, Meiying Wu, Ling-feng Min

**Affiliations:** 1 Department of Tuberculosis, Suzhou Fifth People’s Hospital (Affiliated Infectious Disease Hospital of Soochow University), Suzhou, China; 2 Department of Respiratory and Critical Care Medicine, Northern Jiangsu People’s Hospital (Affiliated to Clinical Medical College of Yangzhou University), No. 98 Nantong West Road, Yangzhou, 225000 Jiangsu People’s Republic of China

**Keywords:** Endoplasmic reticulum stress, Pulmonary tuberculosis, Bioinformatics analysis, Diagnostic markers, Tuberculosis

## Abstract

Tuberculosis (TB), caused by Mycobacterium tuberculosis (MTB), is a serious infectious disease. Previous studies have shown that endoplasmic reticulum (ER) stress plays an important role in various infectious processes. This study aimed to identify potential ER stress-related genes in TB by analyzing differentially expressed genes (DEGs) and to explore the role of ER stress in MTB infection. Ten ER stress-related DEGs (ERSRDEGs) were identified by standardizing and analyzing differential expression in the dataset GSE114911. Gene Ontology (GO) and Kyoto Encyclopedia of Genes and Genomes (KEGG) enrichment analyses revealed that these ERSRDEGs are significantly involved in neutrophil migration and the *IL-17* signaling pathway. A protein-protein interaction network identified four hub genes (*IL-1B*,* CCL20*,* IL -1 A*,* and TNF*), among which gene *IL-1B* showed highly significant differential expression in the independent dataset GSE147964, demonstrating excellent diagnostic performance (AUC = 0.93). This finding was further validated by Enzyme-linked immunosorbent assay (ELISA), which confirmed high expression of *IL-1B* in the plasma of TB patients. Immune infiltration analysis showed increased infiltration of M1 macrophages in the TB infection group, with gene *IL-1B* strongly positively correlated with M1 macrophage abundance. Additionally, the study analyzed correlations between genes *IL-1 A* and *IL-1B* and clinical indicators, including inflammatory factors and D-dimer levels. The results indicated that gene *IL-1B* was positively correlated with *IL-6*, *TNF-α*, and *IFN-γ*, while gene *IL-1 A* was positively correlated with D-dimer. Overall, these findings emphasize the critical role of ER stress-related genes in the pathophysiology of MTB infection.

**Supplementary Information:**

The online version contains supplementary material available at 10.1038/s41598-025-29599-7.

## Introduction

 Tuberculosis (TB), caused by Mycobacterium tuberculosis (MTB), is a serious infectious disease that has long posed a significant threat to human health, primarily due to the widespread prevalence of MTB infection^1^. In 2023, there were 10.8 million new cases of TB globally, resulting in 1.25 million deaths, with profound implications for public health and economic stability. Despite the availability of effective anti-TB treatment regimens, management strategies have become increasingly complex due to the prolonged duration of therapy, significant side effects, and the emergence of multidrug-resistant (MDR) strains. These factors underscore the importance of in-depth research into the pathogenesis of TB, particularly regarding host immune responses and the identification of potential biomarkers for early diagnosis. Growing evidence suggests that the occurrence and progression of TB are closely associated with dysregulated endoplasmic reticulum stress (ERS), which plays a significant role in the disease’s pathophysiological processes.

When the accumulation of misfolded proteins within cells exceeds the capacity of the endoplasmic reticulum (ER) to maintain homeostasis, it triggers ERS and activates a cellular response known as the unfolded protein response (UPR)^2^. Pathogen-induced ERS has been widely reported in infections caused by bacteria, viruses, fungi, and protozoan parasites, including TB^3^. Previous studies have identified associations between ER stress and various inflammatory diseases, highlighting its potential relevance in TB pathogenesis^4^. First reported in 2010, most genes related to ER stress were found to be upregulated in MTB granulomas in both humans and mice^5^. Subsequently, Mycobacterium bovis, Mycobacterium smegmatis, and Mycobacterium avium have also been shown to induce ERS during infection^5^. However, the specific role of ER stress and its associated genes in TB hasnot been thoroughly elucidated. Investigating the expression profiles of ER stress-related genes (ERSRGs) during MTB infection may provide new insights into disease pathogenesis and inform the development of novel therapeutic strategies^6^.

To address this knowledge gap, this study employs bioinformatics methods to conduct an in-depth analysis of publicly available datasets, with a particular focus on the expression profiles of ER stress-related differentially expressed genes (DEGs; ERSRDEGs) in TB patients. By integrating data from the GEO database, our goal is to identify differentially expressed ERSRDEGs in TB patients compared to healthy controls. By correlating gene expression data with functional enrichment analyses and protein-protein interaction (PPI) networks, we aim to elucidate the complex molecular mechanisms linking ERS and TB infection. In summary, our study seeks to pave the way for innovative diagnostic and therapeutic strategies to address this ongoing global health threat^7^.

## Materials and methods

### Data download

The dataset GSE114911 (pulmonary TB caused by MTB) and the dataset GSE147964^8^ (used as the validation set) were downloaded from the GEO database^9^ (https://www.ncbi.nlm.nih.gov/geo/). All samples in both datasets were derived from Homo sapiens. Detailed sample information is provided in Table S1. All M. TB-infected samples and normal control samples were included in this study.

Endoplasmic reticulum stress related genes(ERSRGs)were collected from the GeneCards database (https://www.genecards.org/)^10^ and the MSigDB database (https://www.gsea-msigdb.org/gsea/msigdb).Detailed information is provided in Table S1)^11^. The GeneCards database provides comprehensive information on human genes. Using “ERS” as the search term, we identified a total of 2371 ERSRGs (Detailed information is provided in Table S2) after retaining only those annotated as “Protein Coding” and with a relevance score greater than 1. Similarly, the MSigDB database was queried with “Endoplasmic Reticulum Stress” as the keyword, resulting in 2208 ERSRGs. In addition, to supplement and refine the ERSRG gene set, the keywords “Endoplasmic Reticulum Stress-related” and “Endoplasmic Reticulum Stress-associated” were used to search the PubMed database^12-14^ (https://pubmed.ncbi.nlm.nih.gov/), yielding 47 ERSRGs reported in the published literature, After deduplication and integration of all three sources, a total of 3992 ERSRGs were compiled. Detailed information is provided in Table S3.

In the data processing step, we used the R package limma^15^ (version 3.60.3) to standardize the downloaded gene expression data. This process included data annotation, background correction, and normalization. The purpose of standardization was to eliminate systematic errors between experiments and to make gene expression data comparable across different samples. Principal Component Analysis (PCA) was then performed on the expression matrix of dataset GSE114911 to verify the effect of standardization. PCA^16^ is a dimensionality reduction method that extracts feature vectors (principal components) from high-dimensional data and transforms them into low-dimensional representations. These features can be visualized in two-dimensional or three-dimensional plots.

### ERSRDEGs in MTB infection

According to the sample grouping in dataset GSE114911, the samples were divided into an MTB infection (Infected) group and a Normal group. Differential gene expression analysis between the infected and normal groups was performed using the R package limma^15^. A threshold of |logFC| >1 and *p* < 0.05 was set to identify DEGs. Genes with logFC >1 and *p* < 0.05 were considered upregulated DEGs, while genes with logFC < − 1 and *p* < 0.05 were considered downregulated DEGs. The results of the differential analysis were visualized using a volcano plot generated with the R package ggplot2 (version 3.5.1).

To identify ERSRDEGs associated with MTB infection, all DEGs from dataset GSE114911 were intersected with the ERSRG list, and a Venn diagram was generated to visualize the overlap. The R package pheatmap (version 1.0.12) was used to create a heatmap of the ERSRDEGs. Additionally, the R package RCircos^17^ was employed to generate a Circos plot for further visualization of the genomic distribution of these genes.

### Gene ontology (GO) and pathway (KEGG) enrichment analysis

GO analysis^18^ is a commonly used method for conducting large-scale functional enrichment studies, including the categories of Biological Process (BP), Cellular Component (CC), and Molecular Function (MF). The Kyoto Encyclopedia of Genes and Genomes (KEGG)^19^ is a widely used database that stores information about genomes, biological pathways, diseases, and drugs. We performed GO and KEGG enrichment analyses of the ERSRDEGs using the R package clusterProfiler^20^. The entry screening criteria were adjusted *p* < 0.05 and a false discovery rate (FDR, q-value) < 0.25, with p-value correction conducted using the Benjamini-Hochberg (BH) method.

### Gene set enrichment analysis (GSEA)

GSEA^21^ is used to evaluate the distribution trends of gene expression data to determine their contribution to specific phenotypes. In this study, the genes in dataset GSE114911 were first ranked according to their logFC values. The R package clusterProfiler^20^ was then used to perform GSEA on all genes in the dataset. The gene set employed for GSEA was the C2 curated gene sets from the Molecular Signatures Database (MSigDB) (file name: c2.all.v2023.1.Hs.symbols.gmt), which encompass a broad collection of biological pathways and literature-compiled signaling pathways suitable for systematic enrichment analysis. The parameters for the GSEA were as follows: the random seed was set to 2024, the minimum number of genes per gene set was 10, and the maximum was 500. The screening criterion for significance was adjusted *p* < 0.05, with p-value correction performed using the BH method.

### PPI network

The PPI network comprises proteins that interact with each other and participate in various biological processes (BP), including signal transduction, gene expression regulation, energy and substance metabolism, and cell cycle control. Systematic analysis of protein interactions is of great significance for understanding how proteins function within biological systems, elucidating the mechanisms of signal transduction and metabolic regulation under physiological and pathological conditions, and clarifying functional relationships among proteins. In this study, the STRING database^22^ (https://cn.string-db.org/) was used to construct the PPI network based on the identified ERSRDEGs, applying a minimum interaction score threshold of 0.4. Closely connected regions within the PPI network may represent molecular complexes with specific biological functions. Genes in the PPI network that exhibited interactions with other genes were selected for subsequent analyses. The network was visualized using Cytoscape^23^ software.

All five algorithms from CytoHubba^24^, namely Maximal Clique Centrality (MCC), Degree, Maximum Neighborhood Component (MNC), Edge Percolated Component (EPC), and Closeness, were applied to evaluate the network. The scores of the ERSRDEGs were calculated using the Closeness algorithm, and the top five ERSRDEGs were selected based on these scores. Finally, the genes identified by each of the five algorithms were intersected and analyzed using a Venn diagram. The genes present in the intersection of all algorithms were designated as ER stress-related hub genes.

The GeneMANIA database^25^ enables the identification of functionally similar genes by leveraging a large collection of genomics and proteomics data. In this mode, GeneMANIA assigns weights to each functional genomic dataset based on its predictive value for the query genes. Another application of GeneMANIA is gene function prediction: given a query gene, the platform identifies genes that are likely to share similar functions based on their interaction patterns. In this study, we used the GeneMANIA online tool to predict genes functionally related to the identified hub genes and to construct a PPI network.

### Construction of regulatory network

Transcription factors (TFs) regulate gene expression by interacting with hub genes at the post-transcriptional level. Using the ChIPBase database^26^, the mRNA-TF regulatory network was constructed and visualized with Cytoscape software.

In addition, microRNAs (miRNAs) play important regulatory roles in biological development and evolution. To analyze the relationships between hub genes and miRNAs, the StarBase v3.0 database^27^ was used to construct the mRNA-miRNA regulatory network, which was also visualized in Cytoscape.

Long non-coding RNAs (lncRNAs) similarly have critical regulatory functions in development and can modulate the expression of multiple target genes. To explore the interactions among hub genes, miRNAs, and lncRNAs, the StarBase v3.0 database was employed to build the competing endogenous RNA (ceRNA) regulatory network, which was visualized using Cytoscape software.

### Validation of differential expression of hub genes and receiver operating characteristic (ROC) curve analysis

To further investigate differences in the expression of hub genes between the MTB-infected group and the normal group in datasets GSE114911 and GSE147964, group comparison plots were generated based on hub gene expression levels. Finally, the R package pROC^28^ was used to plot ROC curves for the hub genes and calculate the area under the curve (AUC) to evaluate their diagnostic performance in detecting M. TB infection. The AUC of an ROC curve typically ranges between 0.5 and 1. An AUC closer to 1 indicates better diagnostic accuracy. Specifically, an AUC between 0.5 and 0.7 suggests low accuracy, an AUC between 0.7 and 0.9 indicates moderate accuracy, and an AUC greater than 0.9 reflects high diagnostic performance.

### Validation of clinical specimens

Between December 2024 and February 2025, peripheral blood samples were collected from patients undergoing TB screening at the Fifth People’s Hospital of Suzhou.

In the process of sample inclusion and grouping in this study, the research subjects were strictly screened in accordance with the established criteria. The inclusion criteria for ATB and HC samples included: Patients with ATB are diagnosed based on clinical signs, such as cough, hemoptysis, loss of weight, chest pain, fever, night sweat, and shortness of breath, with bacteriological evidence by any of smear, culture or Xpert from sputum, and the absence of anti-tuberculosis treatment; HC definition: No tuberculosis symptoms, comorbidities, negative T-SPOT/PPD, normal blood indices, and unremarkable chest radiography. Each research participant signed an informed consent form and agreed to participate in the study. The exclusion criteria included: the study subjects suffering from autoimmune diseases; receiving any immunosuppressive drug treatment; being positive for HIV test and having malignant tumor. According to clinical signs, bacteriological evidence and IGRA test, these samples were divided into two groups: ATB group (40cases), and HC group (40 cases). Through the above inclusion and exclusion criteria, it was ensured that the case group and the control group had good homogeneity in demographic characteristics, minimizing the interference of relevant demographic variables on the molecular expression results to the greatest extent. The main purpose of this study is to compare the differences in molecular expression between diagnosed pulmonary tuberculosis patients and healthy controls, focusing on exploring the molecular mechanisms related to the disease. The Ethics Committee of Fifth People’s Hospital of Suzhou has granted approval for the conduct of this study, ensuring its ethical compliance and scientific validity (Ethical approval no. Approval No.: ZF-2024-008-01).

### ELISA

The PPI network analysis identified four core genes, among which genes *IL-1A*and *IL-1B* represented the intersection of the mRNA-miRNA, mRNA-TF, and ceRNA regulatory networks. Additionally, gene *IL-1B* demonstrated a highly significant expression difference in the independent dataset GSE147964 (at the mRNA expression level), along with excellent diagnostic performance (AUC = 0.93). Therefore, we selected genes IL-1 A and IL-1B for ELISA validation (distinct from the aforementioned mRNA expression level analysis).

For this validation(focused on protein level detection), we used 40 blood samples from MTB patients and 40 samples from healthy controls. All patients were recruited from the Fifth People’s Hospital of Suzhou. The candidate biomarkers were assessed using human *IL-1 A* and *IL-1B* ELISA kits (USCN Life Science, Wuhan, China), and all experiments were performed according to the manufacturer’s instructions.

### CIBERSORT immune infiltration analysis

CIBERSORT^29^ is a computational method based on linear support vector regression that deconvolutes transcriptome expression matrices to estimate the composition and abundance of immune cells in mixed cell populations. In this study, the CIBERSORT algorithm, combined with the LM22 signature gene matrix, was applied to dataset GSE114911. Data with immune cell enrichment scores greater than zero were retained, and the resulting immune cell infiltration matrix was obtained. A proportional bar chart was generated to visualize the distribution of immune cell types. Subsequently, the R package ggplot2 (version 3.5.1) was used to create group comparison plots illustrating the differences in immune cell proportions between the infected and normal groups. Immune cell types that showed significant differences between groups were identified for further analysis. The correlations among immune cells were calculated using Spearman’s rank correlation, and the R package pheatmap (version 1.0.12) was used to generate a correlation heatmap displaying these relationships. Finally, the correlations between hub genes and immune cells were also assessed using Spearman’s method, and correlation bubble plots were created with ggplot2 to visualize the associations between hub gene expression and immune cell infiltration.

### Statistical analysis

All data processing and analyses in this study were performed using R software (version 4.4.0). For comparisons of continuous variables between two groups, the statistical significance of normally distributed variables was assessed using independent Student’s t-tests, unless otherwise specified. The Mann-Whitney U test (Wilcoxon rank-sum test) was applied to analyze differences between variables that were not normally distributed. The Kruskal-Wallis test was used for comparisons involving three or more groups. Spearman correlation analysis was performed to calculate correlation coefficients between different genes. All statistical p-values were two-sided unless otherwise indicated, and a p-value of less than 0.05 was considered statistically significant.

## Results

### Standardization of the dataset for MTB infection

First, the R package limma (version 3.60.3) was used to preprocess and standardize the MTB infection dataset GSE114911, including data annotation, background correction, and normalization. The purpose of this standardization was to eliminate systematic errors between experiments, thereby making gene expression data from different samples comparable.

After normalization, we performed PCA on the expression matrix of dataset GSE114911 to evaluate the effectiveness of the normalization process. PCA is a widely used dimensionality reduction technique that can effectively extract the primary features from high-dimensional data.

In the PCA plot generated from the standardized data, samples from the infected and normal groups were clearly separated, indicating significant differences in expression profiles between the groups. The first principal component and the second principal component explained 7.17% and 5.99% of the variance among the samples, respectively, suggesting that the quality of the standardized data was satisfactory and that intergroup differences were pronounced.

**Fig. 1:**
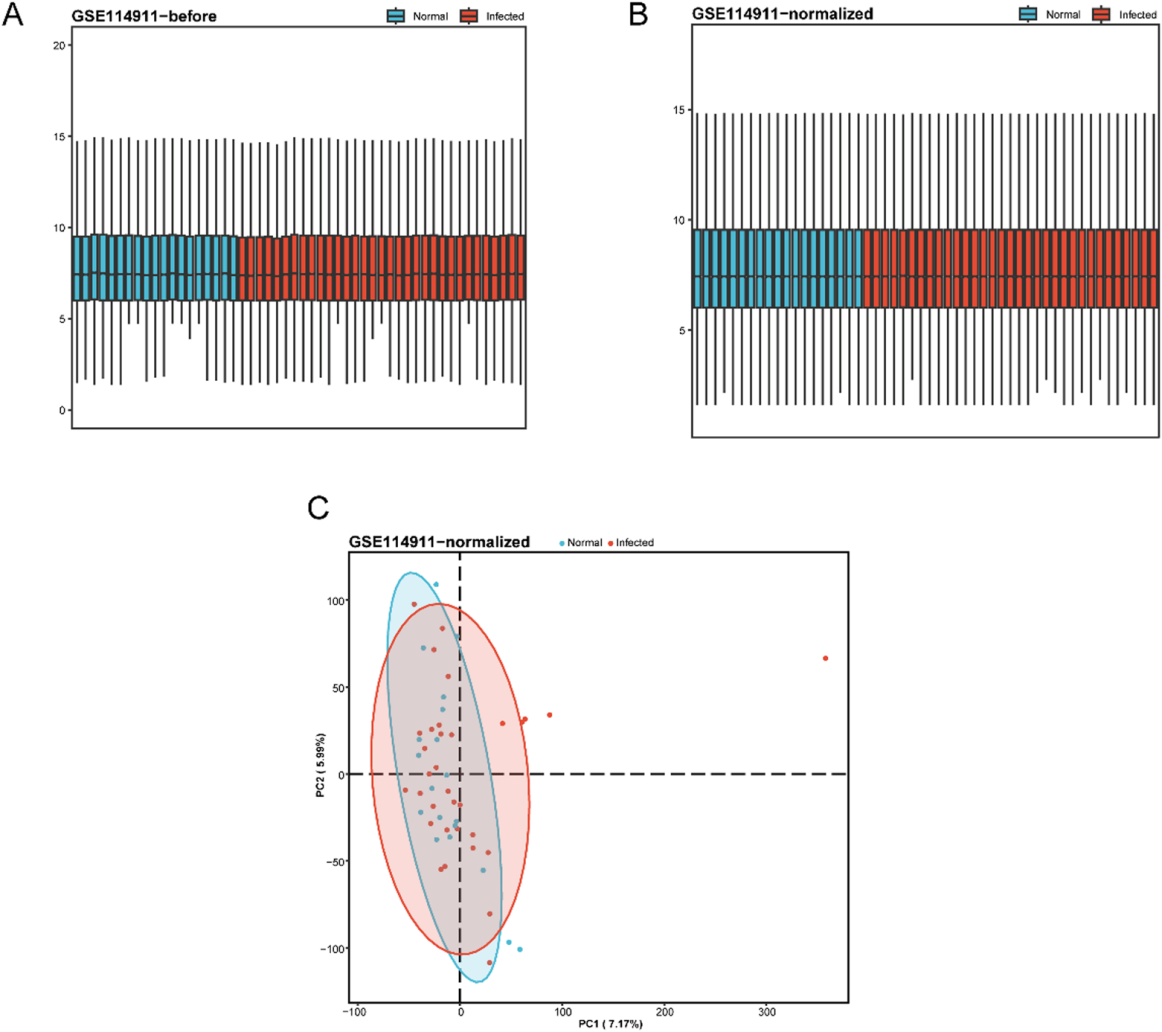
Normalization of GSE114911. (**A**) Boxplot of distribution of GSE114911 dataset before normalization. (**B**) Distribution boxplot of the normalized dataset GSE114911. (**C**) PCA plot of the normalized dataset GSE114911. PCA, Principal Component Analysis. Infected samples with M. tuberculosis are in red, and normal samples are in blue.

Distribution boxplots were used to compare the datasets before and after standardization (Fig. 1A,B), while PCA plots illustrated the principal component distributions of the samples prior to and following normalization (Fig. 1C). The results demonstrated that the data distribution after standardization was more uniform and regular (Table 1).

**Table 1:** GEO microarray chip Information.

	GSE114911	GSE147964
Platform	GPL6480	GPL23126
Species	Homo sapiens	Homo sapiens
Tissue	lung tissue	blood samples
Samples in Infected group	33	10
Samples in Normal group	19	10
Reference	PMID: 29,977,236	

*GEO* gene expression omnibus.

### ERSRDEGs associated with MTB infection

**Fig. 2:**
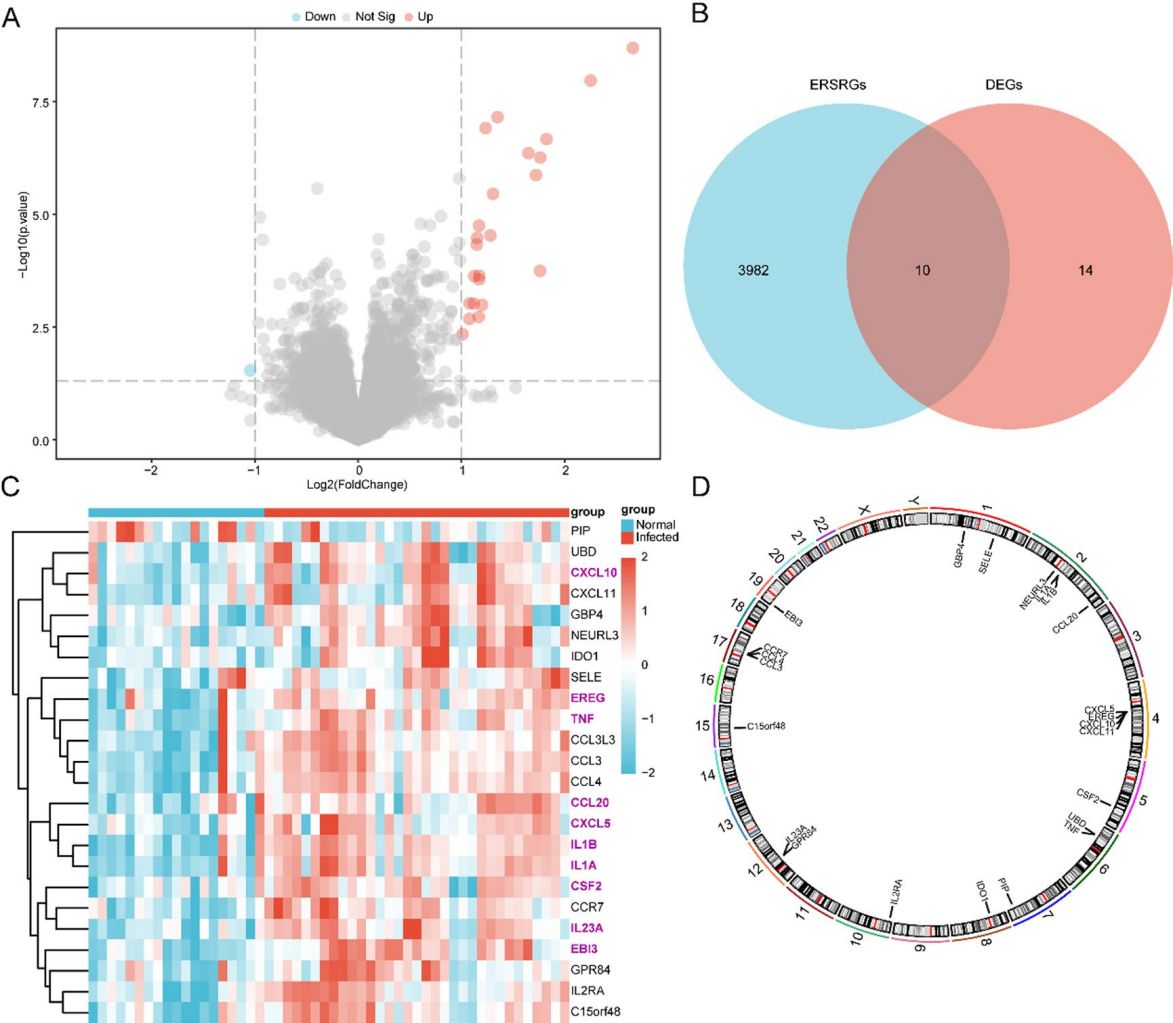
Differential gene expression analysis. (**A**) Volcano plot showing the DEGs between the MTB-infected group and the normal group in dataset GSE114911. (**B**) Venn diagram illustrating the intersection of DEGs and ERSRGs. (**C**) Heatmap of ERSRDEGs in dataset GSE114911. Light red indicates the MTB-infected group, and light blue indicates the normal group. In the heatmap, red represents high expression, and blue represents low expression. (**D**) Chromosomal mapping of ERSRDEGs across the human genome.

The data from dataset GSE114911 were divided into the MTB-infected group and the normal group. We analyzed differences in gene expression values between these two groups and identified 23 upregulated DEGs and 1 downregulated DEG. A volcano plot was generated to visualize the results of the differential expression analysis (Fig. 2A).

To identify ERSRDEGs, all DEGs were intersected with the set of ERSRGs, and a Venn diagram was generated (Fig. 2B). A total of 10 ERSRDEGs were identified. Based on these intersection results, expression differences between the infected and normal sample groups were analyzed, and a heatmap was created using the R package pheatmap to visualize ERSRDEG expression patterns (Fig. 2C). Finally, the chromosomal locations of the ERSRDEGs were analyzed with the R package RCircos, and a chromosome localization plot was generated (Fig. 2D). The chromosomal mapping showed that ERSRDEGs were distributed across chromosomes 1, 2, 4, 5, 6, 7, 8, 10, 12, 15, 17, and 19. The hub genes (see Sect. 2.6) were primarily concentrated on chromosome 2, which contained three genes: *IL-1 A*, *IL-1B*, and *CCL20*. The remaining hub gene, *TNF*, was located on chromosome 6.

### GO and KEGG enrichment analysis

**Fig. 3:**
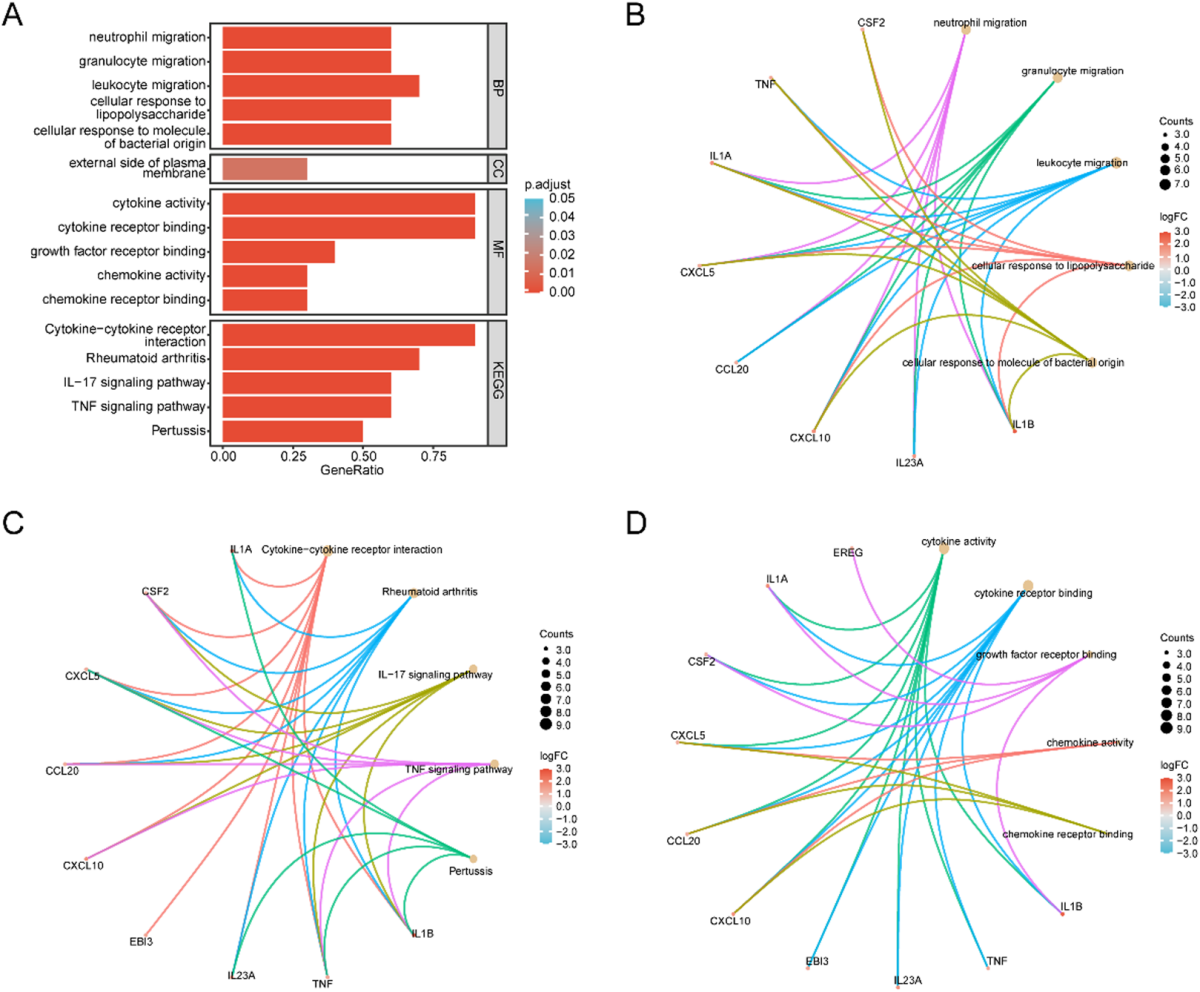
GO and KEGG enrichment analysis for ERSRDEGs. (**A**) Bar graph of GO and KEGG enrichment analysis results for ERSRDEGs. (**B**–**D**) Network diagrams showing GO and KEGG enrichment of ERSRDEGs: BP (**B**), MF (**C**), and KEGG pathways (**D**). In the network diagrams, light brown nodes represent functional terms, and node size indicates the number of genes enriched in each term. Numbers within the nodes correspond to the gene count. Edges (lines) represent the associations between genes and their annotated functions. The larger the functional node, the more genes are enriched in that pathway or term. The color of gene nodes reflects the log fold change (logFC), with red indicating upregulated genes and blue indicating downregulated genes. In all network diagrams, lines connect genes to their associated functional entries. The legend indicates the number of genes represented by each node. The relatively small number of ERSRDEGs is due to the strict differential analysis criteria, underscoring the ERSRGs most closely linked to TB infection. In the bar graph, the color of each column represents the adjusted p-value (adj.p), where red corresponds to smaller adj.p values and blue to larger ones. GO and KEGG screening criteria were adj.p < 0.05 and FDR (q-value) < 0.25, with p-value correction performed using the BH method.

The 10 ERSRDEGs were subjected to GO and Kyoto KEGG pathway enrichment analyses. The detailed results are presented in Table 2. The GO enrichment analysis revealed that these genes were primarily involved in BP such as neutrophil migration, granulocyte migration, leukocyte migration, cellular response to lipopolysaccharide, and cellular response to molecules of bacterial origin. In terms of CC, the genes were mainly enriched in the external side of the plasma membrane. Regarding MF, the enriched terms included cytokine activity, cytokine receptor binding, growth factor receptor binding, chemokine activity, and chemokine receptor binding. KEGG pathway analysis showed enrichment in cytokine-cytokine receptor interaction, rheumatoid arthritis, *IL-17* signaling pathway, T-cell receptor signaling pathway, and pertussis-related pathways. The enrichment analysis results were visualized using bar plots (Fig. 3A).

**Table 2:** Results of GO and KEGG enrichment analysis for ERSRDEGs.

Ontology	ID	Description	GeneRatio	BgRatio	pvalue	padj	qvalue
BP	GO:1,990,266	neutrophil migration	6/10	129/18,888	1.85E-11	2.13E-08	5.83E-09
BP	GO:0097530	granulocyte migration	6/10	154/18,888	5.45E-11	3.13E-08	8.57E-09
BP	GO:0050900	leukocyte migration	7/10	396/18,888	1.92E-10	7.36E-08	2.01E-08
BP	GO:0071222	cellular response to lipopolysaccharide	6/10	225/18,888	5.40E-10	1.49E-07	4.07E-08
BP	GO:0071219	cellular response to molecule of bacterial origin	6/10	238/18,888	7.57E-10	1.49E-07	4.07E-08
CC	GO:0009897	external side of plasma membrane	3/10	405/19,894	9.04E-04	1.45E-02	1.05E-02
MF	GO:0005125	cytokine activity	9/10	238/18,522	8.12E-17	2.52E-15	8.55E-16
MF	GO:0005126	cytokine receptor binding	9/10	273/18,522	2.84E-16	4.41E-15	1.50E-15
MF	GO:0070851	growth factor receptor binding	4/10	138/18,522	5.98E-07	6.18E-06	2.10E-06
MF	GO:0008009	chemokine activity	3/10	49/18,522	2.06E-06	1.60E-05	5.42E-06
MF	GO:0042379	chemokine receptor binding	3/10	74/18,522	7.20E-06	4.46E-05	1.52E-05
KEGG	hsa04060	Cytokine-cytokine receptor interaction	9/10	298/8848	4.81E-13	3.80E-11	1.27E-11
KEGG	hsa05323	Rheumatoid arthritis	7/10	94/8848	1.42E-12	5.63E-11	1.87E-11
KEGG	hsa04657	IL-17 signaling pathway	6/10	95/8848	2.65E-10	6.98E-09	2.32E-09
KEGG	hsa04668	TNF signaling pathway	6/10	119/8848	1.05E-09	2.07E-08	6.90E-09
KEGG	hsa05133	Pertussis	5/10	78/8848	1.14E-08	1.80E-07	5.99E-08

GO, gene ontology, BP, Biological Process; CC, Cellular Component; MF, Molecular Function; KEGG, Kyoto Encyclopedia of Genes and Genomes; ERSRDEGs, Endoplasmic Reticulum Stress Related Differentially Expressed Genes.

Additionally, network diagrams of the enrichment analysis results were generated (Fig. 3B–D). Because only a single term was enriched for the CC category, no network diagram was created for this aspect. In the diagrams, edges indicate the relationships between genes and their corresponding annotation terms. The size of each pathway node reflects the number of genes enriched within that pathway. The color of each gene node represents the log fold change (logFC), with red indicating upregulated genes and blue indicating downregulated genes.

### GSEA enrichment analysis

**Fig. 4:**
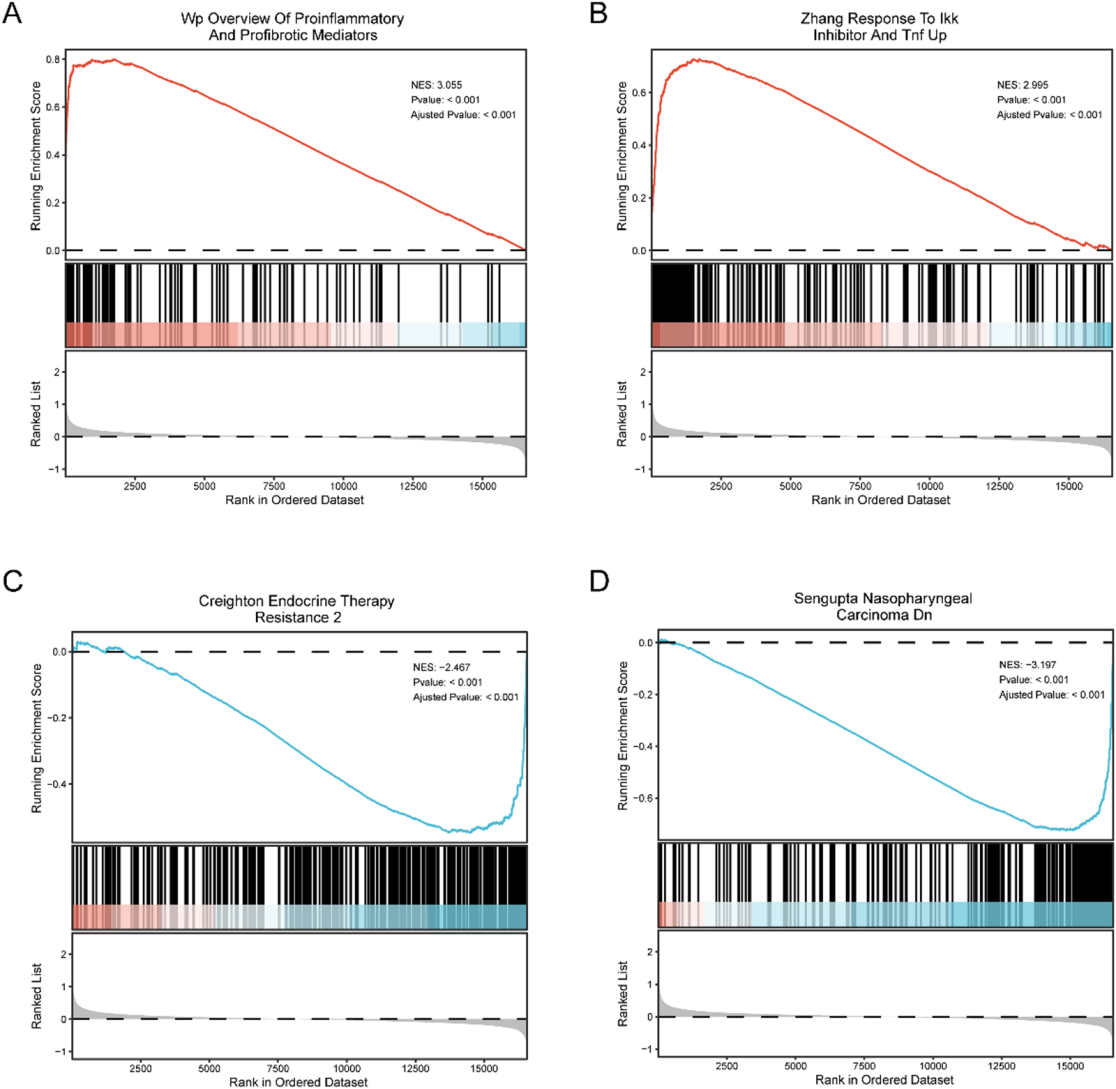
GSEA for GSE114911. (**A**–**D**) GSEA results for dataset GSE114911. Upregulated genes were significantly enriched in OVERVIEW OF PROINFLAMMATORY AND PROFIBROTIC MEDIATORS (**A**) and ZHANG RESPONSE TO IKK INHIBITOR AND T UP (**B**), among other biologically relevant functions and signaling pathways. Downregulated genes were significantly enriched in CREIGHTON ENDOCRINE THERAPY RESISTANCE 2 (**C**) and NEUT SENGUPTA NASOPHARYNGEAL CARCINOMA DN (**D**). GSEA was performed using adjusted p-value < 0.05 and FDR (q-value) < 0.25 as the screening criteria. p-value correction was applied using the BH method.

To evaluate the effects of gene expression levels in dataset GSE114911 on MTB infection, GSEA was performed to assess the associations between gene expression and related BP, CC, and MF. Detailed results are presented in Table 3. The analysis revealed that upregulated genes in dataset GSE114911 were significantly enriched in pathways and functions such as OVERVIEW OF PROINFLAMMATORY AND PROFIBROTIC MEDIATORS (Fig. 4A) and ZHANG RESPONSE TO IKK INHIBITOR AND T UP (Fig. 4B), among other biologically relevant processes and signaling pathways. In contrast, downregulated genes were significantly enriched in pathways including CREIGHTON ENDOCRINE THERAPY RESISTANCE 2 (Fig. 4C) and NEUT SENGUPTA NASOPHARYNGEAL CARCINOMA DN (Fig. 4D), as well as additional related biological functions.

**Table 3:** Results of GSEA for GSE114911.

ID	Set size	Enrichment score	NES	*p* value	*p* adjust	q value
WP_OVERVIEW_OF_PROINFLAMMATORY_AND_PROFIBROTIC_MEDIATORS	117	0.7997	3.0553	1.00E-10	1.09E-08	8.89E-09
ZHANG_RESPONSE_TO_IKK_INHIBITOR_AND_TNF_UP	207	0.7260	2.9950	1.00E-10	1.09E-08	8.89E-09
LINDSTEDT_DENDRITIC_CELL_MATURATION_A	59	0.8635	2.9564	1.00E-10	1.09E-08	8.89E-09
BLANCO_MELO_COVID19_SARS_COV_2_INFECTION_CALU3_CELLS_UP	301	0.6827	2.9426	1.00E-10	1.09E-08	8.89E-09
SANA_TNF_SIGNALING_UP	79	0.8076	2.9329	1.00E-10	1.09E-08	8.89E-09
BLANCO_MELO_HUMAN_PARAINFLUENZA_VIRUS_3_INFECTION_A594_CELLS_UP	182	0.7147	2.9014	1.00E-10	1.09E-08	8.89E-09
ALTEMEIER_RESPONSE_TO_LPS_WITH_MECHANICAL_VENTILATION	117	0.7508	2.8687	1.00E-10	1.09E-08	8.89E-09
KEGG_CYTOKINE_CYTOKINE_RECEPTOR_INTERACTION	251	0.6774	2.8632	1.00E-10	1.09E-08	8.89E-09
SEKI_INFLAMMATORY_RESPONSE_LPS_UP	72	0.8002	2.8464	1.00E-10	1.09E-08	8.89E-09
REACTOME_INTERLEUKIN_10_SIGNALING	44	0.8719	2.8319	1.00E-10	1.09E-08	8.89E-09
SENGUPTA_NASOPHARYNGEAL_CARCINOMA_DN	275	-0.7248	-3.1974	1.00E-10	1.09E-08	8.89E-09
CREIGHTON_ENDOCRINE_THERAPY_RESISTANCE_2	337	-0.5475	-2.4669	1.00E-10	1.09E-08	8.89E-09
KEGG_LYSOSOME	118	-0.5861	-2.3168	1.85E-10	1.82E-08	1.49E-08
KEGG_MEDICUS_VARIANT_MUTATION_CAUSED_ABERRANT_SOD1_TO_RETROGRADE_AXONAL_TRANSPORT	27	-0.7365	-2.2351	2.87E-06	0.000102	8.35E-05
SANA_TNF_SIGNALING_DN	80	-0.5863	-2.1766	2.70E-07	1.26E-05	1.03E-05
LAIHO_COLORECTAL_CANCER_SERRATED_DN	79	-0.5787	-2.1434	2.34E-07	1.10E-05	9.02E-06
FOROUTAN_INTEGRATED_TGFB_EMT_DN	70	-0.5864	-2.1412	1.24E-06	4.95E-05	4.05E-05
APPEL_IMATINIB_RESPONSE	31	-0.6844	-2.1296	1.44E-05	0.000402	0.000329
RICKMAN_HEAD_AND_NECK_CANCER_D	32	-0.6709	-2.0959	4.06E-05	0.000974	0.000797
FOROUTAN_TGFB_EMT_DN	102	-0.5334	-2.0735	5.10E-07	2.17E-05	1.77E-05

GSEA, Gene Set Enrichment Analysis; NES, Normalized Enrichment Score.

Figure 3 shows the results of GO and Kyoto KEGG enrichment analyses conducted on the selected ERSRDEGs, highlighting the functional enrichment of these specific genes in BP and signaling pathways. In contrast, Fig. 4 presents the GSEA results based on the entire gene expression dataset GSE114911, illustrating the overall enrichment trends of all expressed genes across different biological pathways. While Fig. 3 focuses on the functional classification and pathway associations of DEGs, Fig. 4 emphasizes the enrichment trends of the complete gene expression profile. Therefore, the analytical focus and methodological approaches of the two Figs. differ accordingly.

### Construction of protein-protein interaction network and screening of hub genes

**Fig. 5:**
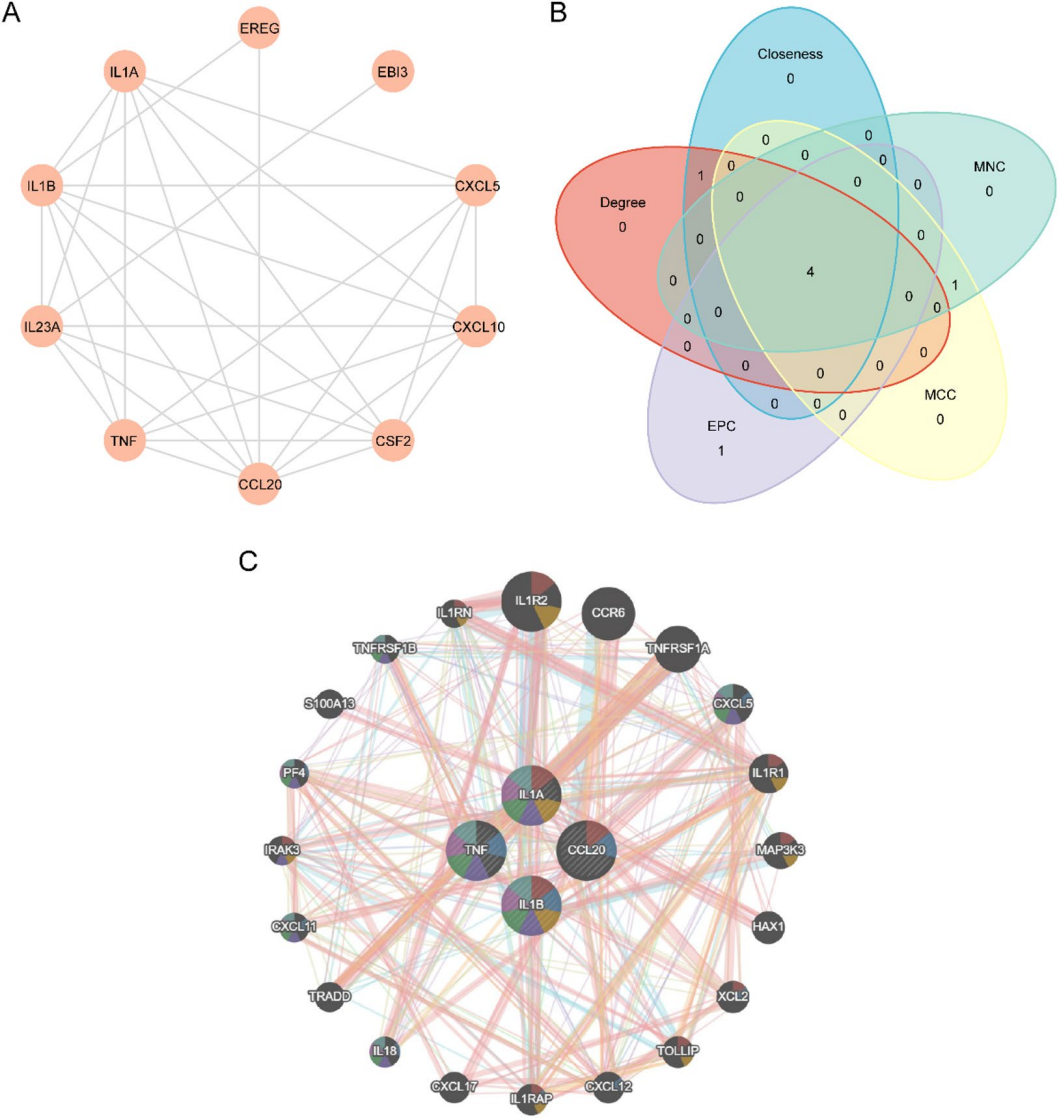
PPI network analysis for ERSRDEGs. (**A**) PPI Network of ERSRDEGs calculated from STRING database. (**B**) Intersection Venn diagram of the top 5 genes with scores of 10ERSRDEGs calculated by MCC, Degree, MNC, EPC and Closeness algorithms of CytoHubba plugin. (**C**) GeneMANIA website predicted the interaction network of functionally similar genes of ERSRDEGs. The circles in the figure show the studied ERSRDEGs and their functionally similar genes, and the corresponding colors of the lines represent the interconnected functions. PPI, Protein-protein Interaction Network; ERSRDEGs, Endoplasmic Reticulum Stress Related Differentially Expressed Genes; MCC, Maximal Clique Centrality; MNC, Maximum Neighborhood Component; EPC, Edge Percolated Component.

First, a PPI network of the 10 ERSRDEGs was constructed using the STRING database and visualized with Cytoscape software (Fig. 5A). The PPI network demonstrated that all 10 ERSRDEGs were interconnected.

In this study, hub genes refer to key genes identified by intersecting the results of multiple ranking algorithms applied to the ERSRDEGs. Specifically, we evaluated the 10 ERSRDEGs using five algorithms implemented in the CytoHubba plugin in Cytoscape: MCC, Degree, MNC, EPC, and Closeness. For each algorithm, the top five ranked genes were selected, and the intersection of these gene sets was determined. A Venn diagram was generated to illustrate the overlap among the algorithms (Fig. 5B), where the colors of the circles indicate different scoring methods. Through this process, four hub genes were identified: *IL-1B*, *CCL20*, *IL-1 A*, and *TNF*. These hub genes were subsequently prioritized in further bioinformatics analyses and experimental validation as core molecules potentially involved in the pathogenesis and progression of TB.

Finally, the interaction network of the four hub genes and their functionally similar genes was predicted and constructed using the GeneMANIA platform (Fig. 5C). In the network diagram, edges of different colors represent co-expression relationships and shared functional information, such as common protein domains. The network included the four hub genes and 20 additional functionally similar proteins.

### Construction of regulatory networks

**Fig. 6:**
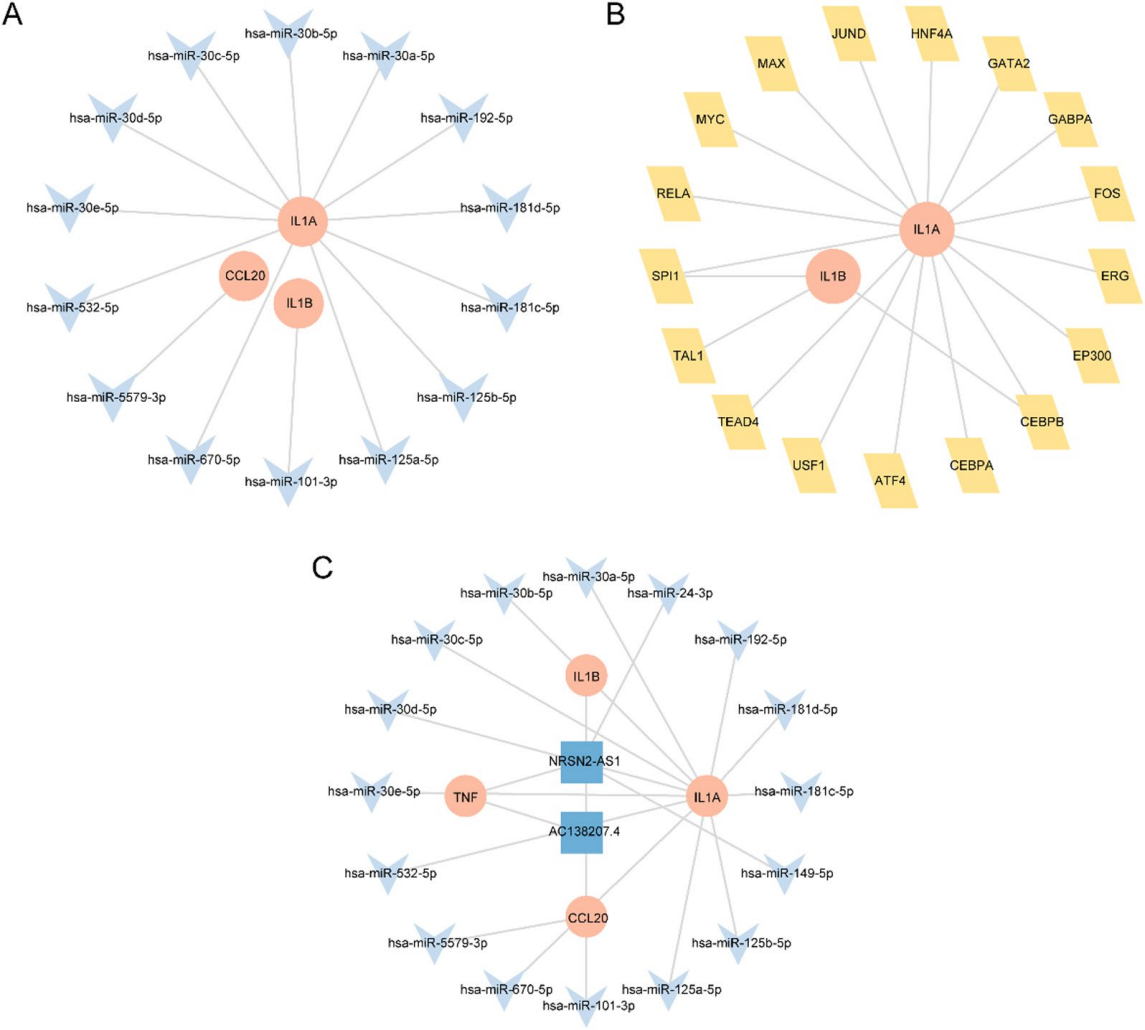
Regulatory network of hub genes. (**A**) mRNA-miRNA Regulatory Network of Hub Genes. (**B**) mRNA-TF Regulatory Network of Hub Genes. (**C**) ceRNA Regulatory Network of Hub Genes (Hub Genes) orange is mRNA, light blue is miRNA, light yellow is TF, dark blue is lncRNA. miRNA, microRNA; TF, Transcription factors; ceRNA, competing endogenous RNA, lncRNA, long non-coding RNA.

After identifying the hub genes, we further explored their upstream and downstream regulatory mechanisms. First, miRNAs associated with the hub genes (*IL-1B*,* CCL20*,* IL-1 A*,* and TNF*) were retrieved from the StarBase database. An mRNA-miRNA regulatory network was then constructed and visualized using Cytoscape software (Fig. 6A). The resulting network included three hub genes and 14 miRNAs. Detailed information about these interactions is provided in Table 4.

**Table 4:** mRNA-miRNA interaction of hub genes.

mRNA	miRNA
CCL20	hsa-miR-5579-3p
IL-1 A	hsa-miR-30a-5p
IL-1 A	hsa-miR-192-5p
IL-1 A	hsa-miR-30c-5p
IL-1 A	hsa-miR-30d-5p
IL-1 A	hsa-miR-181c-5p
IL-1 A	hsa-miR-30b-5p
IL-1 A	hsa-miR-125b-5p
IL-1 A	hsa-miR-125a-5p
IL-1 A	hsa-miR-30e-5p
IL-1 A	hsa-miR-181d-5p
IL-1 A	hsa-miR-532-5p
IL-1 A	hsa-miR-670-5p
IL-1B	hsa-miR-101-3p

miRNA, microRNA.

Next, TFs interacting with the hub genes were identified using the ChIPBase database. An mRNA-TF regulatory network was constructed and visualized (Fig. 6B). This network comprised two hub genes and 19 TFs. Detailed information about these interactions is provided in Table 5.

**Table 5:** mRNA-TF interaction of hub genes.

mRNA	TF
IL-1 A	ATF4
IL-1 A	CEBPA
IL-1 A	CEBPB
IL-1 A	EP300
IL-1 A	ERG
IL-1 A	FOS
IL-1 A	GABPA
IL-1 A	GATA2
IL-1 A	HNF4A
IL-1 A	JUND
IL-1 A	MAX
IL-1 A	MYC
IL-1 A	RELA
IL-1 A	SPI1
IL-1 A	TEAD4
IL-1 A	USF1
IL-1B	SPI1
IL-1B	TAL1
IL-1B	CEBPB

TF, Transcription Factors.

Finally, lncRNAs associated with the hub genes (*IL-1B*,* CCL20*,* IL-1 A*,* and TNF*), as well as lncRNAs related to the corresponding miRNAs, were retrieved from the StarBase database. A ceRNA regulatory network was then constructed (Fig. 6C). This network included four hub genes, 16 miRNAs, and two lncRNAs.

### Differential expression verification and ROC curve analysis of hub genes

**Fig. 7:**
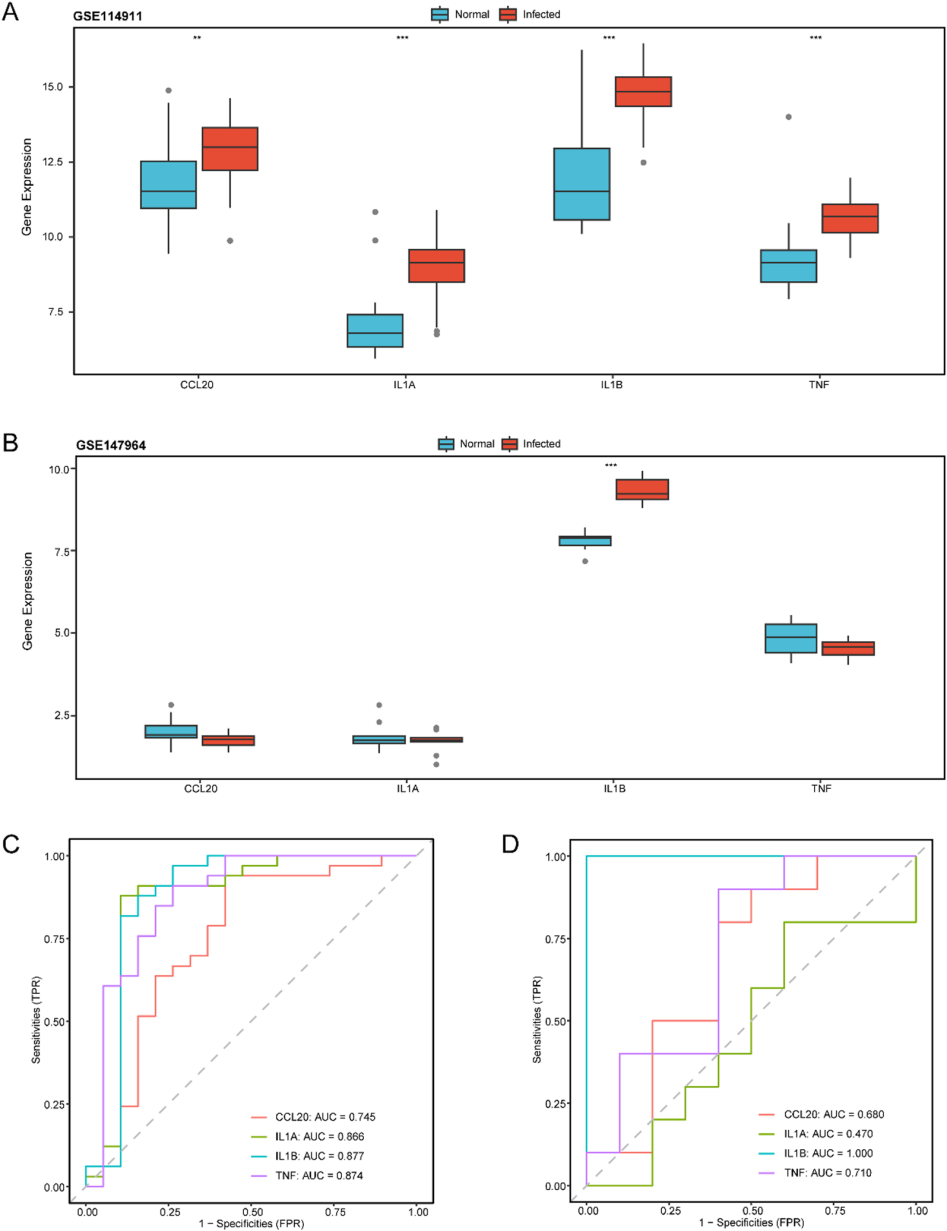
Differential expression validation and ROC curve analysis. (**A**) Cluster comparison diagram of Hub Genes in dataset GSE114911 of Mycobacterium tuberculosis infection (Infected) group and Normal group. (**B**) The grouping comparison of Hub Genes in the Mycobacterium tuberculosis infection (Infected) group and the Normal group in the dataset GSE147964. C-D. ROC curves of four Hub Genes (Hub Genes) in dataset GSE114911 (**C**) and dataset GSE147964 (**D**). ** stands for p value < 0.01, highly statistically significant; *** represents p value < 0.001 and extremely statistically significant. When AUC > 0.5, it indicates that the expression of the molecule is a trend to promote the occurrence of the event, and the closer the AUC is to 1, the better the diagnostic effect. AUC had low accuracy in the range of 0.5 to 0.7, and AUC had moderate accuracy in the range of 0.7 to 0.9. ROC, Receiver Operating Characteristic; AUC, Area Under the Curve; TPR, True Positive Rate; FPR, False Positive Rate. Group comparison boxplots (**A**,**B**), red represents the MTB infection (Infected) group and blue represents the Normal (Normal) group.

To explore and validate the differential expression of hub genes in MTB infection, group comparison analyses were performed. The comparison plots (Fig. 7A,B) show the expression levels of the four hub genes in the Infected and Normal groups in datasets GSE114911 and GSE147964, respectively. In dataset GSE114911, the expression levels of all four hub genes were significantly different between groups (*p* < 0.05).Detailed information is provided in Table S4. In dataset GSE147964, only one hub gene, *IL-1B*, showed a statistically significant difference in expression between the Infected and Normal groups (*p* < 0.05). Finally, the R package was used to generate ROC curves based on the expression levels of the hub genes in both datasets. In dataset GSE114911 (Fig. 7C), the expression of all four hub genes demonstrated moderate classification accuracy for distinguishing MTB-infected samples from controls (0.7 < AUC < 0.9). In dataset GSE147964 (Fig. 7D), the expression of *CCL20* showed low accuracy (0.5 < AUC < 0.7), *TNF* showed moderate accuracy (0.7 < AUC < 0.9), and *IL-1B* demonstrated high diagnostic accuracy (AUC > 0.9) for differentiating MTB infection from normal samples.

### Validation of potential biomarkers expression through ELISA

**Fig. 8:**
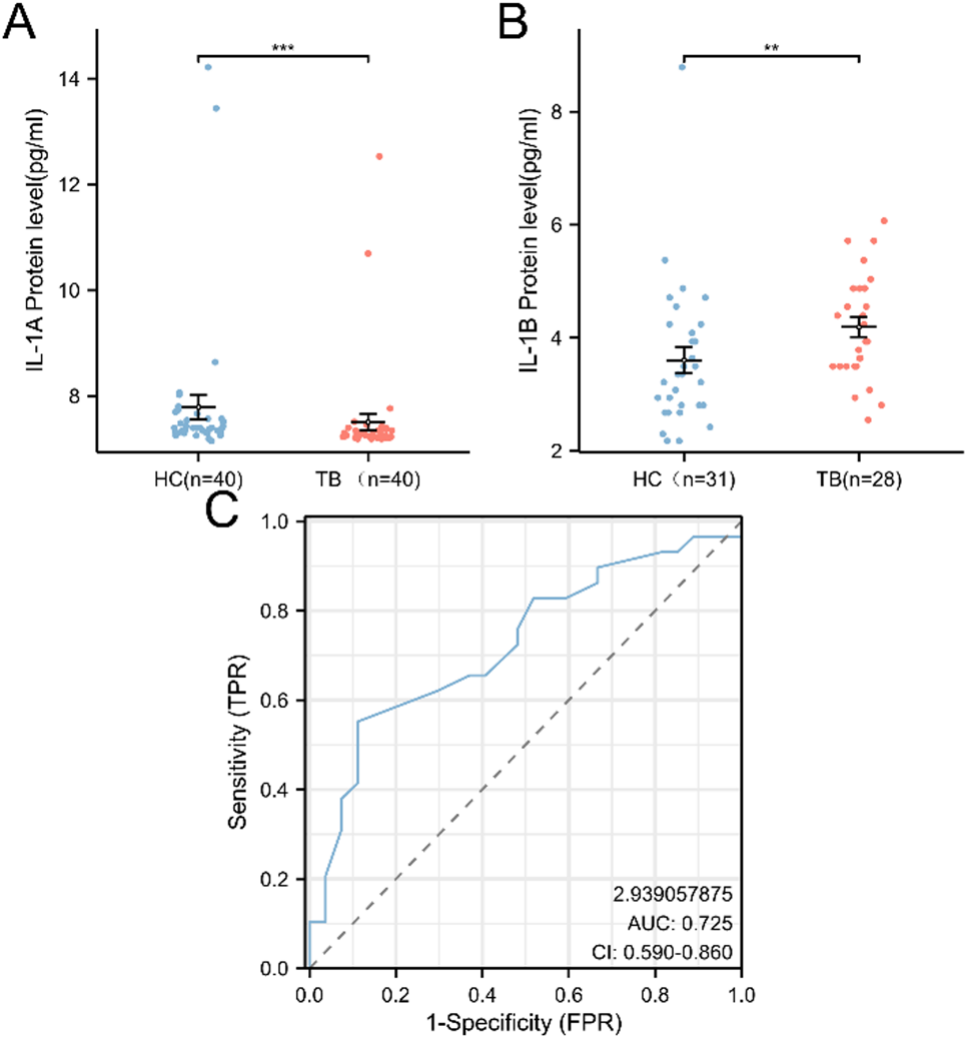
Results of *IL-1 A* and *IL-1B* ELISA and the ROC curve of *IL-1B*. (**A**) The protein levels of IL-1A in the plasma of tuberculosis patients and healthy controls; (**B**) The protein levels of IL-1B in the plasma of tuberculosis patients and healthy controls; (**C**) ROC curve of IL-1B; ** represents p value < 0.01; *** represents p value < 0.001; HC means healthy control, TB means tuberculosis.

The PPI network identified four core genes, among which *IL-1 A* and *IL-1B* were the intersection of the mRNA-miRNA, mRNA-TF, and ceRNA regulatory networks. In datasets GSE114911 and GSE147964, the expression levels of *IL-1B* were significantly upregulated in TB patients compared to the healthy control group. Notably, in the independent dataset GSE147964, the expression difference of *IL-1B* was highly significant and demonstrated excellent diagnostic performance (AUC = 0.93). Based on these findings, *IL-1 A* and *IL-1B* were selected for clinical validation. The experimental results confirmed that *IL-1B* was upregulated in the TB group, consistent with the bioinformatics analysis (Fig. 8A,B). However, the ROC curve generated from the clinical validation data showed an AUC of 0.725 (Fig. 8C), indicating that *IL-1B* may serve as a potential diagnostic biomarker for TB.

### Analysis of the correlation between the expression levels of ERS-related genes and clinical indicators

**Fig. 9:**
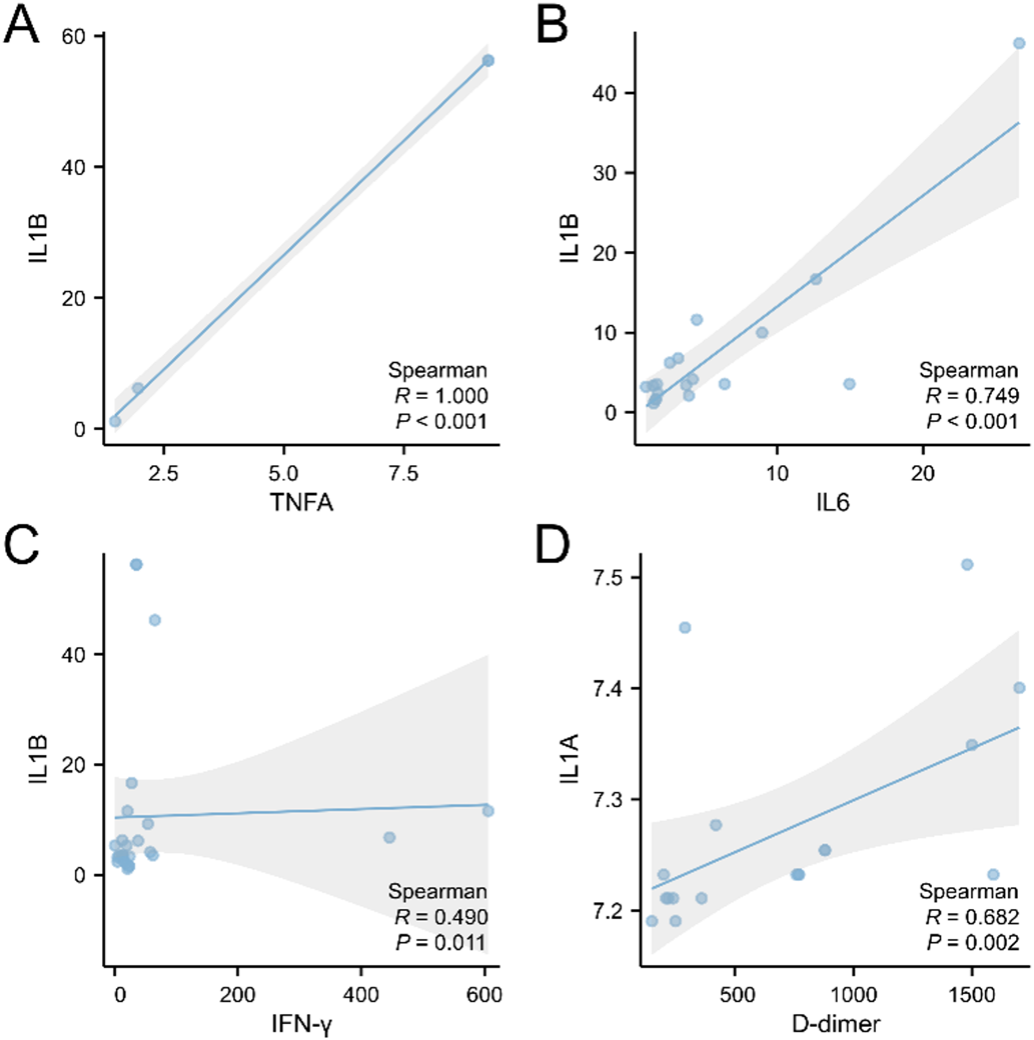
Correlation analysis of *IL-1 A* and *IL-1B* with clinical indicators. (**A**) IL-1B is positively correlated with IL-6. (**B**) IL-B is positively correlated with TNFA. (**C**) IL-1B is positively correlated with IFN-γ. (**D**) IL-1 A is positively correlated with D-dimer.

Statistical analysis was performed on patient-related clinical test reports *(IL-6*, *TNF-α*, *IFN-γ*, and D-dimer). The inflammatory factors *IL-6*,* TNF-α*, and *IFN-γ* were theoretically assessed at the protein level using flow cytometry. The correlation between the expression levels of ER stress-related genes and clinical indicators (inflammatory factors and D-dimer) was analyzed, as shown in Fig. 9. The results demonstrated that *IL-1B* was positively correlated with *IL-6*, *TNF-α*, and *IFN-γ* (Fig. 9A-C). Additionally, A exhibited a significant positive correlation with D-dimer (*P* < 0.05) (Fig. 9D).

### CIBERSORT immune infiltration analysis

**Fig. 10:**
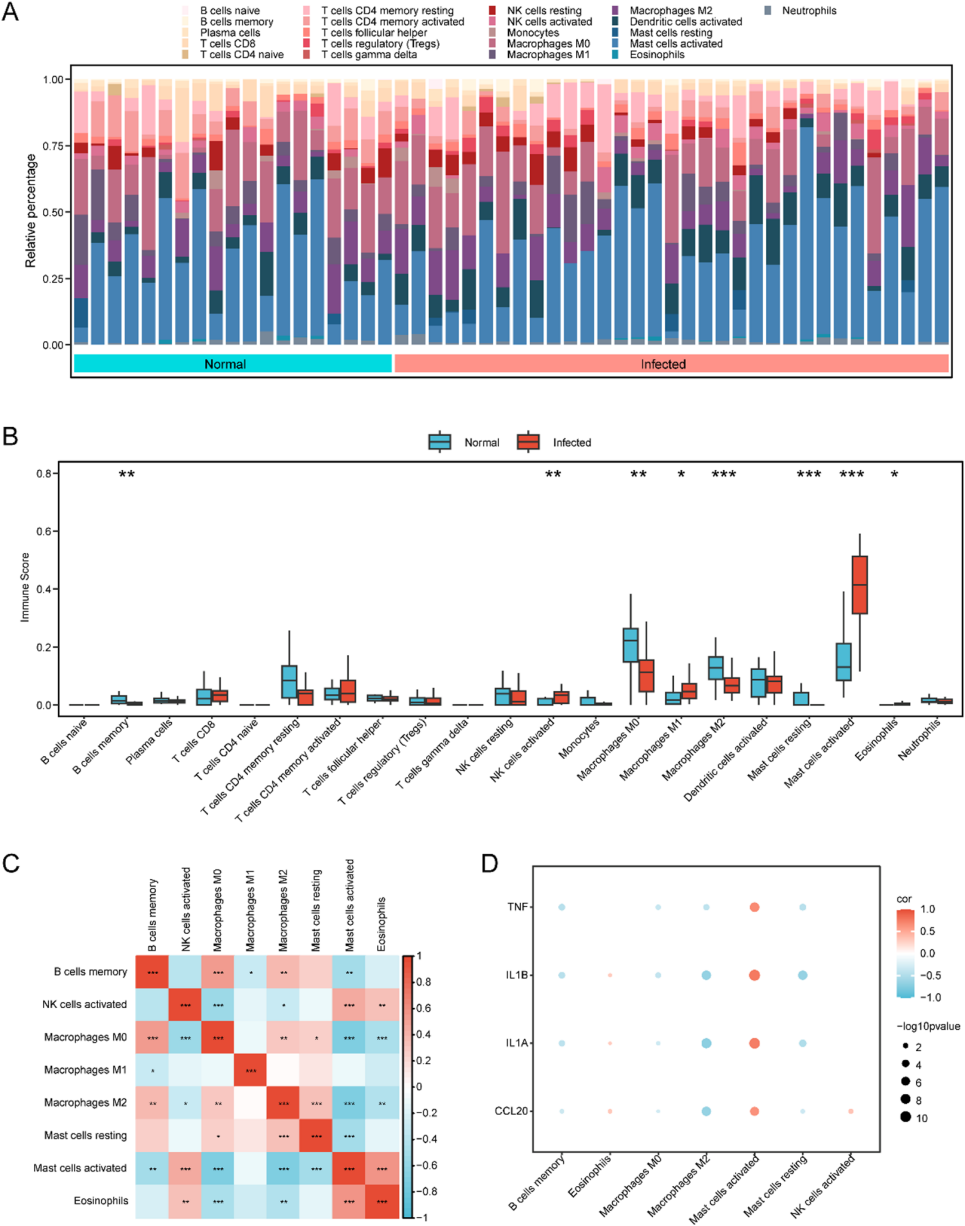
Immune infiltration analysis by CIBERSORT algorithm. (**A**) Bar chart of the abundance of immune infiltration in the Infected group and the Normal group of the dataset GSE114911. (**B**) The grouping comparison of immune cell infiltration abundance in the Mycobacterium tuberculosis infection (Infected) group and the Normal group in dataset GSE114911. (**C**) Correlation heatmap of immune cells in immune cell infiltration abundance matrix. (**D**) Bubble plot of correlation between Hub Genes and immune cell infiltration abundance in dataset GSE114911. * represents p value < 0.05, indicating statistical significance; ** represents p value < 0.01, highly statistically significant; *** represents p value < 0.001 and extremely statistically significant. The light red is the Mycobacterium tuberculosis Infected group, and the light blue is the Normal group. Red is positive correlation, blue is negative correlation. The depth of the color represents the strength of the correlation.

According to the results of the immune infiltration analysis, a bar chart illustrating the proportions of immune cell types was generated (Fig. 10A). Next, immune cell types with p values < 0.05 were identified through group comparison analysis, highlighting significant differences in infiltration abundance between the Infected and Normal groups. The group comparison plot (Fig. 10B) showed that eight immune cell types, memory B cells, activated NK cells, M0 macrophages, M1 macrophages, M2 macrophages, resting mast cells, activated mast cells, and eosinophils, were significantly different between the groups (*p* < 0.05). Subsequently, the correlation matrix of all 22 immune cell types in the GSE114911 dataset was visualized as a correlation heatmap (Fig. 10C). Finally, the correlations between the hub genes and the eight significantly altered immune cell types were assessed and presented as a correlation bubble plot (Fig. 10D).

## Discussion

TB is an infectious disease caused by MTB and remains a major threat to global health, affecting millions of people each year^[30. The^ disease primarily manifests as pulmonary TB, leading to severe respiratory complications and contributing to high morbidity and mortality rates in low- and middle-income countries^31^.Despite progress in diagnosis and treatment, the emergence of multidrug-resistant strains has posed significant challenges to management strategies, underscoring the urgent need for a deeper understanding of the fundamental biological mechanisms underlying TB pathogenesis and host immune responses^32^. This study aimed to investigate the role of ER stress in TB infection, with a particular focus on the differential expression of ER stress-related genes associated with M. TB. By leveraging publicly available datasets and applying bioinformatics approaches, we identified several key ER stress-related genes that may serve as potential biomarkers for TB. These results not only advance our understanding of the molecular mechanisms of TB but also highlight promising therapeutic targets for improving disease management and patient outcomes.

In the context of MTB infection, the analysis of DEGs provides important insights into the potential molecular mechanisms of the disease. In this study, we identified 24 significantly upregulated DEGs, highlighting the robust inflammatory response triggered by the pathogen. Bioinformatics analysis demonstrated that the expression of *IL-1B* was significantly upregulated in TB patients compared to healthy controls, a finding validated in both the GSE114911 and GSE147964 datasets. Furthermore, ELISA experiments confirmed that the upregulation trend of *IL-1B* in the plasma of TB patients was consistent with the transcriptomic data. It is important to clarify that *IL-1B* is a pro-inflammatory cytokine primarily secreted by immune cells during infection or inflammatory responses. While its upregulation can serve as an indirect marker of cells experiencing ERS, it is not a classical molecule that directly induces ERS. *IL-1B* promotes the expression of inflammatory mediators by activating signaling pathways such as NF-κB and MAPK, thereby contributing to the formation of an inflammatory microenvironment. Studies have shown that *IL-1B* enhances the release of pro-inflammatory factors through these pathways, resulting in a stronger inflammatory response^33^. Consistent with our findings, *IL-1B* was positively correlated with other inflammatory factors, including *IL-6*,* TNF-α*,* and IFN-γ*, and previous research has demonstrated that *IL-1B* can increase the secretion of these cytokines. These mediators, in turn, can activate ERS and the UPR. This inflammatory microenvironment not only contributes to the host’s defense against M. TB infection but also may perpetuate cellular stress responses^34^.

By performing functional pathway enrichment analysis of the DEGs, this study found that they were significantly enriched in key immune-related pathways, including neutrophil migration, cytokine-cytokine receptor interaction, the *IL-17* signaling pathway, and T cell signaling pathways. The enrichment of the neutrophil migration pathway suggests that during TB infection, the rapid recruitment and migration of neutrophils play an important role in limiting pathogen spread and promoting the inflammatory response. Existing studies have shown that neutrophils are critical components of the defense mechanism during the acute phase of TB infection^35^. The significant enrichment of the cytokine-cytokine receptor interaction pathway further highlights the central role of various inflammation-related cytokines (such as *IL-1* and *TNF-α*) and their receptors in regulating host immune responses. The synergistic effects among these molecules are essential for maintaining immune homeostasis and preventing excessive inflammatory damage^36^. In addition, DEGs were significantly enriched in the *IL-17* and T cell signaling pathways. The *IL-17* signaling pathway has been shown to play a key role in regulating chronic inflammation, promoting chemokine expression, and facilitating neutrophil recruitment, directly influencing immune defense and the pathological progression of pulmonary TB^37^. The T cell signaling pathway is indispensable for regulating macrophage activation, granuloma formation, and the maintenance of inflammatory responses, and its dysregulation is closely associated with the progression of Mycobacterium infection.

The results of the CIBERSORT analysis revealed marked changes in immune cell composition in the context of TB infection. Consistent with previous findings^38–41^, significant differences in immune cell abundance were observed between healthy individuals and the active TB (ATB) cohort. Specifically, memory B cells, activated NK cells, M0 macrophages, M1 macrophages, M2 macrophages, resting mast cells, activated mast cells, and eosinophils exhibited statistically significant differences (*p* < 0.05) between the infected and control groups, suggesting that these cell populations may be closely associated with the occurrence and progression of TB. Research has demonstrated that both the number and activity of mast cells in the lung tissue of TB patients are significantly increased, which may be related to infection with MTB and the ensuing inflammatory response^42^. Mast cells detect pathogens through surface receptors and rapidly respond during the early stages of infection by releasing large quantities of inflammatory mediators, thereby regulating the local immune environment^43^. In addition, mast cells influence the activity of T cells and B cells through interactions with other immune cell populations, further shaping the progression and outcome of TB^44^. Consistent with these observations, our results showed that the number of activated mast cells in the TB infection group was significantly higher than in the healthy control group (*p* < 0.05).

The constructed PPI network reveals the complex relationships among these proteins. The significant interactions identified suggest that they may act synergistically to regulate cellular responses to MTB infection^45^.For example, central genes such as *IL-1B* and *CCL20* not only participate in inflammatory signaling but also interact with proteins involved in regulating the ERS pathway, highlighting their multifaceted roles in the infection response ^46^. This network analysis is crucial for elucidating the regulatory mechanisms underlying the pathogenesis of TB and may guide future research aimed at identifying novel therapeutic targets capable of disrupting these interactions^47,48^. Furthermore, understanding the dynamics of the PPI network may facilitate the discovery of potential biomarkers for the diagnosis and monitoring of TB, thereby improving clinical management of the disease^49^.

The occurrence and progression of TB also involve a multilayered molecular regulatory network. In recent years, increasing attention has been directed toward the roles of non-coding RNAs and TFs in regulating TB-related gene expression and ERS processes. miRNAs can participate in diverse BP, including immune regulation, inflammatory responses, and apoptosis, by binding to target mRNAs to inhibit their translation or promote their degradation. For example, multiple studies have confirmed that miR-155, miR-146a, and other miRNAs modulate host immune and inflammatory responses during MTB infection, thereby influencing disease progression^50,51^. The core genes identified in this study, such as *IL-1B* and *CCL20*, have upstream regulatory miRNAs (including miR-101-3p and members of the miR-30 family) that have been reported in the literature to be associated with inflammation and immune status in TB. lncRNAs, as key components of the ceRNA regulatory network, can indirectly influence the stability and translational efficiency of mRNAs by adsorbing miRNAs through a “molecular sponge” effect.

Relevant studies have shown that lncRNAs such as MALAT1 and NEAT1 are abnormally expressed in TB patients and can influence the expression levels of inflammatory factors^52,53^. In this study, the ceRNA regulatory network analysis revealed interactions among lncRNAs, key miRNAs, and mRNAs, suggesting that lncRNAs may regulate the expression of ERS-related genes by modulating miRNA activity, thereby affecting the immune microenvironment and disease progression in TB. TFs, as central regulators of gene expression, also play critical roles in modulating the host response to MTB infection. Multiple TFs, including NF-κB, CEBPB, and SPI1, have been shown to directly bind to the promoter regions of target genes and regulate the transcription of inflammatory mediators^54,55^.In the mRNA-TF regulatory network constructed in this study, core genes such as *IL-1 A* and *IL-1B* were found to be regulated by numerous TFs, further supporting their central role in the occurrence and progression of TB.

ROC curve analysis demonstrates the diagnostic potential of the identified hub genes, highlighting their relevance as biomarkers for TB. The ability of these genes to reliably distinguish between infected and non-infected individuals, combined with their acceptable AUC values, indicates that they could be integrated into diagnostic assays to improve early detection of the disease^56,57^.Early diagnosis is critical for effective TB management, particularly in the context of rising rates of multidrug-resistant strains. Furthermore, the predictive value of these biomarkers may facilitate personalized treatment strategies by enabling the prediction of patient responses to therapy based on individual gene expression profiles. This approach is consistent with the current trend toward precision medicine^58^.

Previous studies have shown that TB can induce a systemic hypercoagulable state, characterized by significantly elevated plasma D-dimer levels, particularly in patients with active or severe disease^59^.This elevation is closely associated with the severity, activity, and prognosis of TB. In the present study, a positive correlation was observed between gene A and D-dimer levels, suggesting that this ERS-related gene may be closely linked to disease severity and prognosis and could serve as a potential prognostic marker for TB.However, the qPCR and ELISA results for gene A were inconsistent with the findings from the bioinformatics training set. This discrepancy may be attributable to sample heterogeneity or the potential suppression of protein expression due to post-translational degradation. Therefore, further studies with larger sample sizes are needed to validate the expression patterns and clinical significance of gene A in TB patients.

In summary, further analysis integrating the key DEGs identified in this study demonstrates that *IL-1B*, *CCL20*, *IL-1 A*, and *TNF* occupy central regulatory positions within the relevant signaling pathways. For example, *IL-1B* is not only significantly upregulated and strongly associated with inflammatory mediator networks but also activates downstream NF-κB and MAPK pathways, thereby amplifying the inflammatory response. *TNF*, as a primary effector molecule in inflammation, directly participates in regulating apoptosis, immune cell migration, and the maintenance of granuloma integrity. *CCL20*, functioning as a chemokine, promotes the directional migration of various immune cells, facilitating the formation of an effective local immune barrier. *IL-1 A* shows a close association with the coagulation state and may influence TB prognosis. The coordinated regulation of these key DEGs across multiple signaling pathways underscores the complex interplay between ERSRGs and immune-inflammatory responses during MTB infection. This systematic analysis, which integrates DEGs with enriched pathway networks, not only advances our understanding of the immunopathogenic mechanisms of TB but also provides a theoretical foundation for the development of novel diagnostic and therapeutic strategies. Future studies, including functional validation experiments and translational clinical research focusing on these core pathways and genes, will be essential to support the precise diagnosis and treatment of TB.

The synergistic regulatory roles of miRNAs, lncRNAs, and TFs in TB and ERS provide a solid theoretical foundation for deepening our understanding of the molecular pathogenesis of TB and for identifying novel diagnostic and therapeutic targets. This study systematically examined the functions of these molecules within the TB regulatory network by integrating bioinformatics analyses with the latest advances reported in the literature. By comparing our findings with recent research in related fields, this work enriches and expands both the depth and breadth of current knowledge. In the future, functional studies and translational clinical applications focusing on miRNAs, lncRNAs, and TFs are expected to offer new strategies and evidence to support the precise diagnosis and treatment of TB.

This study systematically investigated endoplasmic reticulum stress genes associated with Mycobacterium infection and their immunological characteristics by analyzing datasets from lung tissue (GSE114911) and peripheral blood (GSE147964). Lung tissue samples reflect the local immune response at the infection site, revealing distinct cellular compositions, molecular expression profiles, and immune microenvironment features. In contrast, peripheral blood samples represent the systemic immune response, displaying notable differences in cellular subpopulation distribution, activation status, and expression patterns compared with local tissues. Consequently, discrepancies in gene expression and immune dynamics between local (lung) and systemic (blood) responses may lead to inconsistent biomarker performance across sample types. In this study, both the expression and diagnostic value of key genes such as *IL-1B* varied between lung tissue and peripheral blood, underscoring the need to consider expression consistency and potential mechanistic differences across sample sources. Moreover, inherent differences between local and systemic samples limit direct comparison or extrapolation of findings. Therefore, interpretation and clinical application should account for multiple factors, including sample type and disease status.

This study focuses on the molecular mechanisms and marker screening related to Mycobacterium tuberculosis infection. When selecting samples and formulating inclusion and exclusion criteria, key demographic variables (such as age, underlying diseases, etc.) were strictly controlled to ensure the homogeneity of the case group and the control group, thereby reducing the potential interference of demographic characteristics on molecular expression results. This study designed a comprehensive approach using publicly available gene expression databases from non-endemic regions (e.g., GSE114911 and GSE147964) to screen for key genes, combined with molecular validation using clinical samples from endemic regions (high TB ​​prevalence areas). This strategy aims to enhance the generalizability and extrapolation value of the study conclusions.However, immune responses and molecular marker expression vary among populations in different regions, potentially affecting marker sensitivity and specificity and limiting the extrapolation of the results. Thus, regional epidemiological differences should be considered when translating these findings into clinical practice. Future research should include systematic validation across multi-center and multi-population cohorts to evaluate marker stability and broad applicability, thereby enhancing their scientific and clinical value.

Summary: This study reveals the important roles of ER stress-related differentially expressed genes associated with Mycobacterium tuberculosis infection and provides potential biomarkers and targets for future diagnostic and therapeutic strategies. These findings contribute to a better understanding of the mechanisms of Mycobacterium tuberculosis infection and support efforts to address public health challenges.

## Supplementary Information

Below is the link to the electronic supplementary material.

Supplementary Material 1

## Data Availability

The GEO database provides access to the datasets examined in this study (http://www.ncbi.nlm.nih.gov/geo/). Please contact the corresponding author if you have any more questions.
